# ASSOCIATION BETWEEN T CELL SUBSETS AND MORTALITY: FINDINGS FROM THE LONG LIFE FAMILY STUDY

**DOI:** 10.1093/geroni/igae098.2285

**Published:** 2024-12-31

**Authors:** Gokul Seshadri, Sithara Vivek, Mary Wojczynski, Allison Kuipers, Joseph Zmuda, Iva Miljkovic, Svetlana Ukraintseva, Bharat Thyagarajan

**Affiliations:** University of Minnesota, Minneapolis, Minnesota, United States; University of Minnesota, Minneapolis, Minnesota, United States; Washington University in St. Louis, St. Louis, Missouri, United States; University of Pittsburgh, Pittsburgh, Pennsylvania, United States; University of Pittsburgh, Pittsburgh, Pennsylvania, United States; University of Pittsburgh, Pittsburgh, Pennsylvania, United States; Duke University, Durham, North Carolina, United States; University of Minnesota, Minneapolis, Minnesota, United States

## Abstract

Differences in T cell subsets are associated with higher mortality in the general population. We evaluated whether Long Life Family Study (LLFS) participants and their spouses differed from the general population regarding T cell distributions and their association with mortality. We measured 12 T cell subsets in 1647 LLFS offspring (60.77±8.19 years) and 550 spousal controls (61.08±8.57 years). We used a Cox proportional hazards model to evaluate the association between these T cell subsets and 4-year mortality after adjustment for age, sex, family relatedness and CMV seroprevalence. After age adjustment, LLFS offspring and spousal controls had higher levels of CD4+ naïve T cells (27.5% higher) and CD4+ effector memory cells (1600% higher) and lower levels of CD4+ central memory T cells (55% lower) as compared to the Health and Retirement Study (HRS). The difference in CD4+ naïve T cells between those who were < 55 years vs. those > 85 years was 50% lower in LLFS offspring as compared to spousal controls (similar to the HRS controls) (p=0.05). The percentage of CD4+ effector memory cells was associated with increased mortality risk (HR:1.47; p=0.01) and CD4+ naïve T cells showed a borderline inverse association with 4 year mortality (HR: 0.72; p=0.06) in the combined sample of LLFS offspring and spousal controls. These results suggest that levels of T cell subsets are maintained at older ages in long lived families despite CMV exposure and the association between T cells and mortality is similar to what is observed in the general population.

